# High Co-Expression of *PDCD1*/*TIGIT*/*CD47*/*KIR3DL2* in Bone Marrow Is Associated with Poor Prognosis for Patients with Myelodysplastic Syndrome

**DOI:** 10.1155/2023/1972127

**Published:** 2023-02-08

**Authors:** Mengjun Zhong, Cunte Chen, Wenshu Zhao, Jiaxiong Tan, Jie Chen, Xin Huang, Yangqiu Li, Min Dai

**Affiliations:** ^1^Key Laboratory for Regenerative Medicine of Ministry of Education, Institute of Hematology, Jinan University, 510632 Guangzhou, China; ^2^Department of Hematology, Nanfang Hospital, Southern Medical University, 510515 Guangzhou, China; ^3^Department of Hematology, First Affiliated Hospital, Jinan University, Guangzhou 510632, China; ^4^Department of Hematology, Guangdong Provincial People's Hospital (Guangdong Academy of Medical Sciences), Southern Medical University, 510080 Guangzhou, China

## Abstract

Cellular immune disorder is a common characteristic of myelodysplastic syndrome (MDS). Abnormal natural killer (NK) cell function has been reported in MDS patients, and this is closely related to disease progression and poor prognosis. However, little is known about the association between the abnormal immune checkpoint (IC) that results in abnormal immune NK cell function and the prognosis of MDS. In this study, RNA-sequencing data from 80 patients in the GSE114922 dataset and bone marrow (BM) samples from 46 patients with MDS in our clinical center were used for overall survival (OS) analysis and validation. We found that the NK cell-related IC genes *PDCD1*, *TIGIT*, *CD47,* and *KIR3DL2* had higher expression and correlated with poor OS for MDS patients. High expression of *PDCD1* or *TIGIT* was significantly associated with poor OS for MDS patients younger than 60 years of age. Moreover, co-expression of *PDCD1* and *TIGIT* had the greatest contribution to OS prediction. Interestingly, *PDCD1*, *TIGIT*, *CD47*, and *KIR3DL2* and risk stratification based on the Revised International Prognostic Scoring System were used to construct a nomogram model, which could visually predict the 1-, 2-, and 3-year survival rates of MDS patients. In summary, high expression of IC receptors in the BM of MDS patients was associated with poor OS. The co-expression patterns of *PDCD1*, *TIGIT*, *CD47*, and *KIR3DL2* might provide novel insights into designing combined targeted therapies for MDS.

## 1. Introduction

Myelodysplastic syndrome (MDS) is a group of heterogeneous malignancies with distinct natural histories that are characterized by ineffective clonal hematopoiesis, abnormal hematopoietic cell morphology, and varying degrees of cytopenia where one-third of patients progress to acute myeloid leukemia (AML) [1–3]. This disease is more prevalent in older patients aged 65–70 with less than 10% of patients younger than 50 [4, 5]. In elderly patients, the incidence is 7–35 per 10^5^ with men more likely to develop MDS than women [6]. According to the Revised International Prognostic Scoring System (IPSS-R), treatment strategies vary for patients with different risk stratifications [7]. Although chemotherapy, hypomethylating agents, and allogeneic hematopoietic stem cell transplantation have partially benefited patients, MDS treatment still poses a great challenge [8–10]. Multiple factors have been involved in the pathogenesis of MDS, such as cellular immune dysfunction. Previous studies have reported abnormal natural killer (NK) cells in MDS patients with overexpression of immunosuppressive molecules [11], decreased expression of activating NK receptors, reduced antibody-dependent cytotoxicity (ADCC), and lowered direct NK cell lytic function [12]. Moreover, haploidentical NK cell therapy has been reported to achieve complete remission by reducing high-risk MDS clones [13]. Reconstructed NK cells achieve better functional education and help reduce relapse in patients when donors and hosts express all of the KIR ligands for donor KIRs [14]. These findings suggest that targeting NK cell-associated receptors may be a novel immunotherapeutic strategy for MDS patients.

In contrast to T cells, NK cells can migrate to many tissues and initiate immune responses to infections or cancers, and they are able to dissolve certain target cells via cytotoxicity mechanisms that release substances, such as granzymes and perforin, without sensitizing the host [15–17]. Increasing evidence has demonstrated that NK cells are defective in patients with solid tumors [18] or MDS [19], indicating that NK-mediated immune surveillance of tumors may be disrupted, and immunosuppression and immune escape may contribute to disease progression [12, 20]. Recent studies have suggested that immune checkpoint (IC) receptors, such as programmed cell death protein-1 (PD-1), programmed death-ligand 1 (PD-L1), T cell immunoglobulin and ITIM domain (TIGIT), CD47, and cytotoxic T lymphocyte-associated antigen-4 (CTLA-4), are highly expressed in many types of cancer and are currently targeted to improve antitumor responses [21–24]. As the classical immune checkpoint, PD-1 binds to its ligands PD-L1 and programmed death-ligand 2 (PD-L2) to allow tumor cells to evade immune surveillance [25]. In numerous cancers, TIGIT signaling negatively regulates antitumor immunity [26], and CD47 overexpression has been reported to be associated with poor survival and a higher rate of progression to AML in MDS patients [27]. Moreover, cytokines secreted by KIR3DL2 expressing NK cells and T cells could promote the progression of malignances [28]. Our previous study found that dysregulated T cells, caused by low B-cell leukemia/lymphoma 11B (BCL11B) expression, are associated with the prognosis of MDS; however, the effect of abnormal IC receptor expression on NK cells on the clinical outcomes of MDS patients remains unclear [29]. Furthermore, the antitumor effects of IC inhibitors (ICIs) alone are limited, which may be due to heterogeneity in IC receptor expression levels and distinct dominant IC expression patterns in different MDS patients [30, 31]. Therefore, it is worth studying the expression patterns of IC proteins in MDS.

In this study, we investigated the prognostic importance of IC proteins in relation to NK cells using RNA-sequencing data obtained from newly diagnosed MDS patients from the Gene Expression Omnibus (GEO) database, and the results were further validated by quantitative real-time PCR (qRT-PCR) in our clinical center.

## 2. Materials and Methods

### 2.1. GSE114922 Dataset

**Figure 1:**
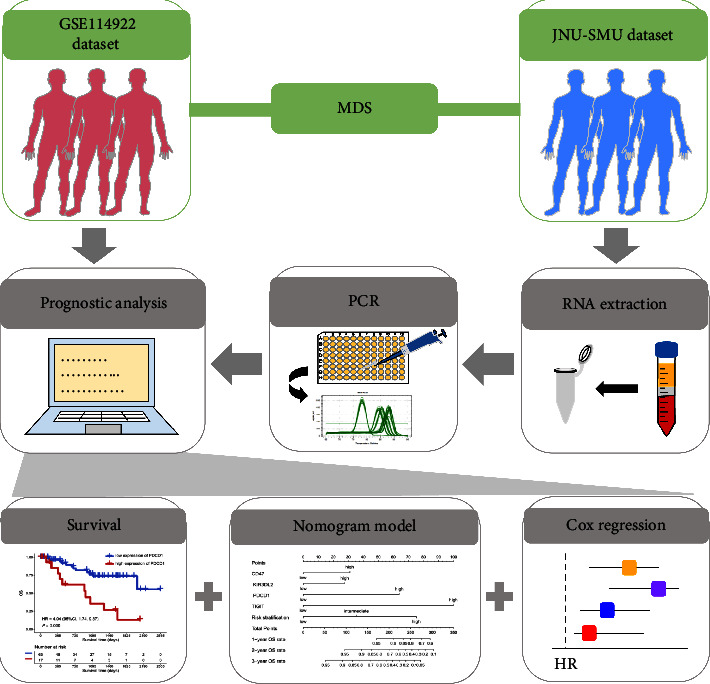
Workflow of study. RNA-sequencing data from 80 patients in the GSE114922 dataset were downloaded from the Gene Expression Omnibus (GEO) database (https://www.ncbi.nlm.nih.gov/geo/) and used for overall survival (OS) analysis, including Kaplan–Meier curve analysis, Cox regression analysis, and a nomogram model. A total of 46 BM samples were collected from newly diagnosed MDS patients from our clinical center for RNA extraction, qRT-PCR, and OS analysis. JNU-SMU, the First Affiliated Hospital of Jinan University and Nanfang Hospital Affiliated to Southern Medical University.

The RNA-sequencing data from 80 de novo MDS patients and corresponding clinical information in the GSE114922 dataset were downloaded from the Gene Expression Omnibus (GEO) database (https://www.ncbi.nlm.nih.gov/geo/). Overall survival (OS) was defined as the time from the date of diagnosis to the date of death or last follow-up. The clinical information of the patients, including age, gender, OS status, cancer type, and risk stratification, is listed in Table S1. The analysis process is shown in Figure 1. Because the GSE114922 dataset is publicly available, approval by a local ethics committee was not required.

### 2.2. BM Samples

A total of 46 BM samples were collected from the newly diagnosed MDS patients at the First Affiliated Hospital of Jinan University and Nanfang Hospital Affiliated to Southern Medical University (JNU-SMU) from March 21, 2017, to March 26, 2020. The median follow-up time was 393 days (range: 27–1,418 days). This study was performed in accordance with the principles of the Declaration of Helsinki and was approved by the Ethics Committee of the First Affiliated Hospital of Jinan University. All participants provided written informed consent.

### 2.3. Quantitative Real-Time PCR (qRT-PCR)

Total RNA was isolated from the BM samples of the MDS patients using TRIzol reagent (Invitrogen, Carlsbad, CA, USA) according to the manufacturer's instructions and was then reverse transcribed into complementary DNA (cDNA) using a reverse transcription kit (Promega Corporation, Madison, Wisconsin, USA) according to the experimental instructions. The relative expression levels of *PDCD1*, *TIGIT*, *CD47*, and *KIR3DL2* were detected by qRT-PCR with SYBR Master Mix (TIANGEN, Beijing, China), and *β*-actin was selected as an internal control. The primer sequences for qRT-PCR are shown in Table S2. The expression levels of *PDCD1*, *TIGIT*, *CD47*, and *KIR3DL2* are presented as 2^−ΔCT^.

### 2.4. Statistical Analysis

All statistical analyses were performed using R (version 4.0.2, https://www.r-project.org/) and Statistical Product and Service Solutions (SPSS) (version 22.0, IBM, Armonk, NY, United States) software. The function “surv_cutpoint” in the R package “survminer” determined the optimal cutoff value for continuous variables (Figures S1 and S2). The log-rank test was used to compare differences in Kaplan–Meier curves. Cox proportional hazards models were constructed with the R package “survival.” A two-tailed *P* value <0.05 was considered to be statistically significant.

## 3. Results

### 3.1. High Expression of *PDCD1*, *TIGIT*, *CD47*, and *KIR3DL2* in the BM of MDS Patients Is Associated with Poor OS

**Figure 2:**
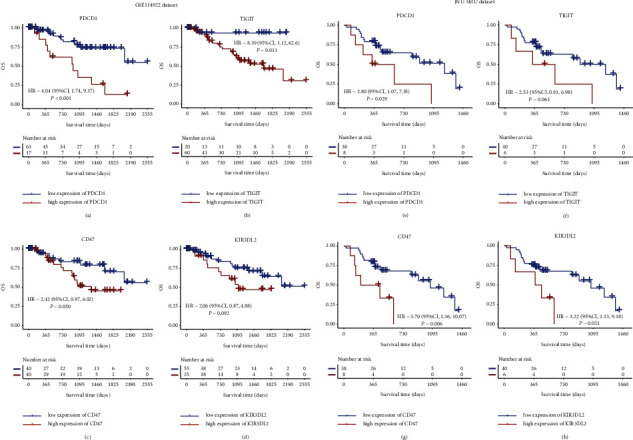
OS analysis of *PDCD1*, *TIGIT*, *CD47*, and *KIR3DL2* for MDS patients. High expression of *PDCD1*, *TIGIT*, *CD47*, and *KIR3DL2* was associated with poor OS. Kaplan–Meier curves were plotted according to groups of high and low expression of *PDCD1* (a), *TIGIT* (b), *CD47* (c), and *KIR3DL2* (d) in the GSE114922 dataset (*n* = 80). OS analysis of *PDCD1* (e), *TIGIT* (f), *CD47* (g), and *KIR3DL2* (h) in the JNU-SMU dataset (*n* = 46).

To investigate the prognostic contribution of NK cell-related IC receptors in MDS patients, we performed survival analysis based on expressions of these genes in the GSE114922 dataset. After the optimal cutoff values for *PDCD1*, *TIGIT*, *CD47*, and *KIR3DL2* were obtained, the patients were divided into low- and high-risk groups (Figures S1 and S2). Interestingly, patients with high expression of *PDCD1*, *TIGIT*, *CD47*, and *KIR3DL2* had poorer OS in the GSE114922 dataset (*PDCD1*: hazard ratio (HR) = 4.04, *P* < 0.001; *TIGIT*: HR = 8.39, *P*=0.013; *CD47*: HR = 2.42, *P*=0.050; *KIR3DL2*: HR = 2.06, *P*=0.092; Figures 2(a)–2(d)). These findings were confirmed in the JNU-SMU dataset (*PDCD1*: HR = 2.80, *P*=0.029; *TIGIT*: HR = 2.53. *P*=0.063; *CD47*: HR = 3.70, *P*=0.006; *KIR3DL2*: HR = 3.22, *P*=0.021; Figures 2(e)–2(h)). These results suggested that high expression of either *PDCD1*, *TIGIT*, *CD47*, or *KIR3DL2* alone could predict poor OS in MDS patients.

### 3.2. *PDCD1*, *TIGIT*, and *CD47* Have Potential for Stratification Prediction in MDS Subgroups

**Figure 3:**
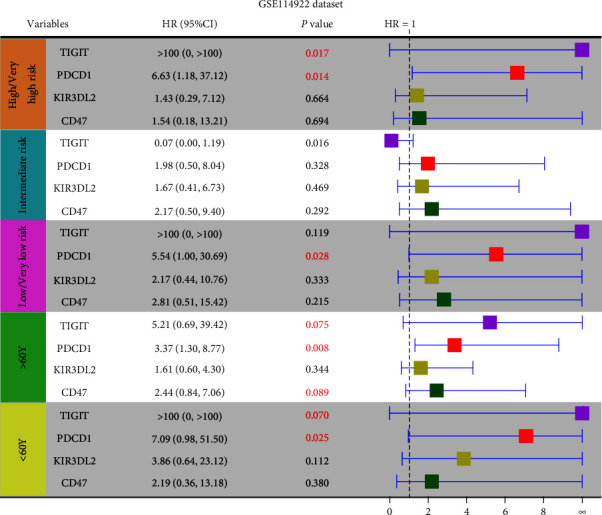
Subgroup analysis of *PDCD1*, *TIGIT*, *CD47*, and *KIR3DL2* for MDS patients in the GSE114922 dataset.

To study the correlation between *PDCD1*, *TIGIT*, *CD47*, and *KIR3DL2* and clinical characteristics, we performed a subgroup analysis using the GSE114922 dataset. As shown in Figure 3, there was a clear trend suggesting that high expression of *PDCD1* or *TIGIT* is associated with poor OS for MDS patients younger than 60 years of age, although the *P* value of *TIGIT* is not statistically significant (*PDCD1*: HR = 7.09, *P* = 0.025; *TIGIT*: HR > 100, *P* = 0.070). Similar results were found for patients older than 60 years, but the *P* values of *TIGIT* and *CD47* are not statistically significant (*PDCD1*: HR = 3.37, *P* = 0.008; *TIGIT*: HR = 5.21, *P* = 0.075; *CD47*: HR = 2.44, *P* = 0.089). In addition, the prognostic value of *PDCD1*, *TIGIT*, *CD47*, and *KIR3DL2* for MDS patients with different risk stratifications was analyzed. Low/very low-risk patients (*n* = 38) with high expression of *PDCD1* had poor OS (HR = 5.54, *P* = 0.028), and high expression of *PDCD1* or *TIGIT* was associated with poor OS for high/very high-risk patients (*n* = 19) (*PDCD1*: HR = 6.63, *P* = 0.014; *TIGIT*: HR > 100, *P* = 0.017). However, there is no significant relationship between the expression of *PDCD1*/*TIGIT*/*CD47*/*KIR3DL2* and OS for intermediate-risk patients (*n* = 19). Moreover, the *KIR3DL2* expression level was not significantly associated with OS in patients with low/very low, intermediate, or high/very high risk (Figure 3). These results indicated that *PDCD1*, *TIGIT*, and *CD47* have potential for stratification prediction in MDS subgroups.

### 3.3. Co-Expression Patterns of *PDCD1*, *TIGIT*, *CD47*, and *KIR3DL2* Have Great Contribution to OS for MDS

**Figure 4:**
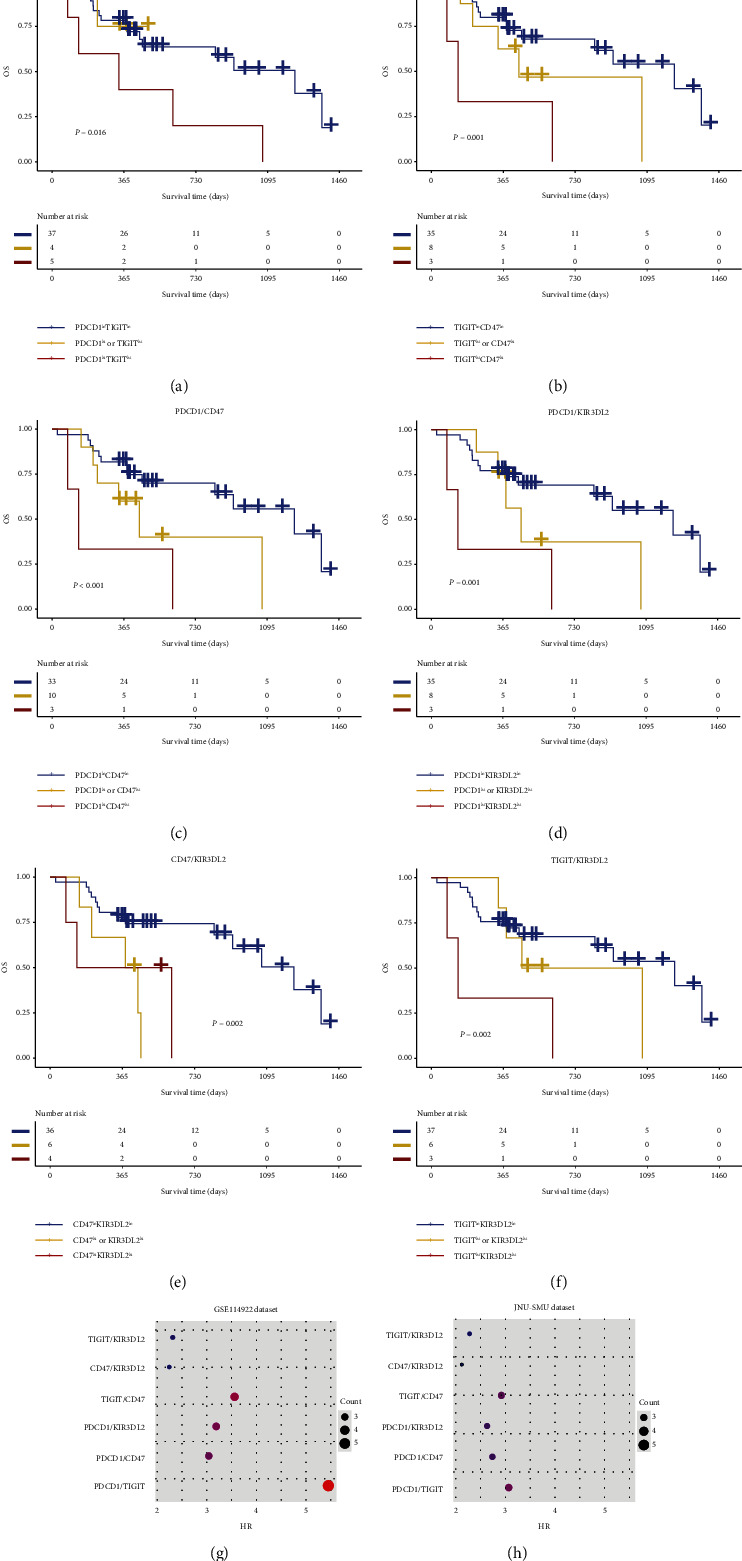
Co-expression patterns of *PDCD1*, *TIGIT*, *CD47*, and *KIR3DL2* in MDS patients. OS analysis of *PDCD1*^high^*TIGIT*^high^ (a), *TIGIT*^high^*CD47*^high^ (b), *PDCD1*^high^*CD47*^high^ (c), *PDCD1*^high^*KIR3DL2*^high^ (d), *CD47*^high^*KIR3DL2*^high^ (e), and *TIGIT*^high^*KIR3DL2*^high^ (f). OS contributions of different co-expression patterns in MDS patients based on hazard ratio (HR) in the GSE114922 (g) and JNU-SMU (h) datasets.

To investigate the effects of different co-expression patterns of *PDCD1*, *TIGIT*, *CD47*, and *KIR3DL2* on the clinical outcomes of MDS patients, we analyzed different combinations of these genes in Kaplan–Meier curves. Significantly, MDS patients who were *PDCD1*_high_*TIGIT*_high_, *TIGIT*_high_*CD47*_high_, *PDCD1*_high_*CD47*_high_, or *PDCD1*_high_*KIR3DL2*_high_ had a worse OS in the GSE114922 dataset (HR > 3, *P* < 0.001) (Figure S3). These results were confirmed in the JNU-SMU dataset (HR > 2, *P* < 0.05) (Figures 4(a)–4(f)). Importantly, to further identify which co-expression pattern has the greatest contribution to OS, we used HR as an evaluation criterion. Interestingly, the top three combinations that contributed to OS prediction were *PDCD1*/*TIGIT*, *TIGIT*/*CD47,* and *PDCD1*/*KIR3DL2*, and *PDCD1*/*TIGIT* had the greatest contribution in the GSE114922 dataset (HR = 5.45, Figure 4(g)). This finding was again confirmed in the JNU-SMU dataset (HR = 3.07, Figure 4(h)).

### 3.4. New Risk Stratification Based on the Nomogram Model Shows Better Performance on OS Prediction

**Figure 5:**
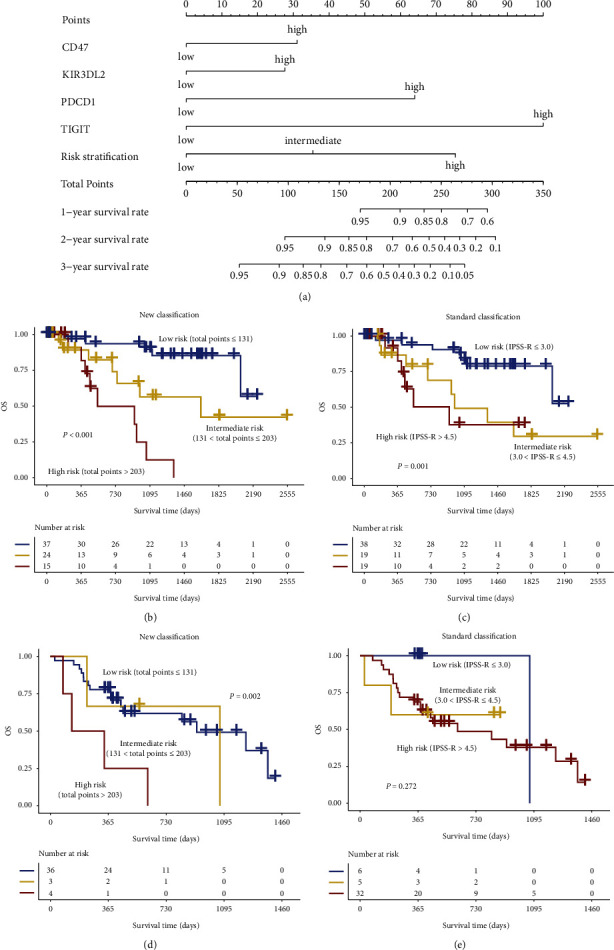
Construction of a nomogram model using the GSE114922 dataset. (a) A nomogram model was constructed according to *PDCD1*, *TIGIT*, *CD47*, and *KIR3DL2* expression and standard risk stratification based on IPSS-R. After a point for *PDCD1*, *TIGIT*, *CD47*, and *KIR3DL2*, the standard risk stratification for each patient was assigned by the nomogram, and the total points could be obtained to predict OS rates. A new risk stratification was constructed from the total points derived from the nomogram model in the GSE114922 (b) and JNU-SMU (d) datasets. Standard risk stratification based on IPSS-R in the GSE114922 (c) and JNU-SMU (e) datasets.

Because standard risk stratification based on IPSS-R and the expression levels of *PDCD1*, *TIGIT*, *CD47*, and *KIR3DL2* were significantly associated with the prognosis of MDS patients, these were all used to construct a nomogram model to visually predict the 1-, 2-, and 3-year survival rates for MDS patients in the GSE114922 dataset (Figure 5(a)). The detailed points and OS rates are shown in Table S3. To provide more precise prognostic prediction for MDS patients, we generated a new risk stratification for patients based on the total points derived from the nomogram model. After obtaining optimal cutoff values 131 and 203 using X-tile software, the patients were divided into low-, intermediate-, and high-risk groups (Figures S4A and S4B). Interestingly, patients with higher risk (total point >203) based on the nomogram model had worse OS than those with low or intermediate risk in the GSE114922 dataset (*P* < 0.001) (Figure 5(b)). This result was also shown in standard risk stratification based on IPSS-R in the GSE114922 dataset where patients with high risk had worse OS compared with those with low or intermediate risk (*P* = 0.001) (Figure 5(c)). Although high-risk patients (total point >203) based on the nomogram model had worse OS than those with low or intermediate risk in the JNU-SMU dataset (*P* = 0.002), standard risk stratification based on IPSS-R was not significantly associated with OS (*P* = 0.272) (Figures 5(d) and 5(e)). Notably, the new risk stratification was an independent predictor for OS in the GSE114922 dataset (HR = 3.51, 95% confidence interval (CI): 1.99 to 6.18, *P* < 0.001). This result was again confirmed in the JNU-SMU dataset (HR = 2.13, 95% CI: 1.20 to 3.78, *P* = 0.002) (Table 1). These findings indicate that the new risk stratification based on the nomogram model had better performance for OS prediction than the standard risk stratification based on IPSS-R.

## 4. Discussion

It has been demonstrated that aberrant expression of IC receptors is significantly associated with NK cell dysfunction in MDS, but little is known about their association with the prognosis of MDS patients [11, 12]. In this study, we found that high expressions of *PDCD1*, *TIGIT*, *CD47*, and *KIR3DL2*, which are related to NK cells, predict poor OS for MDS patients. Moreover, co-expression of *PDCD1*, *TIGIT*, *CD47*, and *KIR3DL2* correlates with poor OS for MDS patients. Of these genes, the co-expression of *PDCD1* and *TIGIT* might be the best OS predictor for MDS. Interestingly, weighted combination of IPSS-R, *PDCD1*, *TIGIT*, *CD47,* and *KIR3DL2* could provide more accurate prognostic stratification for MDS patients.

Harnessing the immune response to target malignant tumors through the use of immune checkpoint blockade (ICB) has now become a vital breakthrough in treating solid tumors [32–34] and hematological malignancies [35]. In this study, we found that overexpression of *PDCD1*, *TIGIT*, *CD47,* and *KIR3DL2* predicted poor OS for MDS patients, and this may indicate that the proteins of these genes might serve as targets for immunotherapy. Nowadays, various clinical trials of ICBs in MDS have been carried out; however, remission could be observed in only a small percentage of MDS patients who were treated with a single dose of monoclonal antibody to ICBs [30, 36–39]. Due to the limited response activities of ICB as a single agent, combination therapy with different ICBs may be used to overcome primary and acquired resistance to single-agent administration and provide more clinical benefit for patients [40]. In a variety of solid tumors, the results of multiple clinical trials of ICB combination therapy for malignant tumors have shown that combining ICBs can lead to higher response rates with manageable safety profiles and good tolerability [41–44]. Primary data from an ongoing Phase II clinical trial suggested that combination of PD-1 and CTLA4 monoclonal antibodies can achieve 58% projected 1-year survival in R/R AML patients [45, 46]. However, clinical trials of ICB combination therapy in MDS patients are recruiting and ongoing, and there are currently no relevant preliminary data [47–50]. Our previous research has found that the combination of two IC proteins was a better predictor for the prognosis for AML patients than individual ones [51]. In this study, we demonstrated that co-expression of *PDCD1* and *TIGIT* may work the best for OS prediction in MDS, which may provide a feasible and effective scheme for ICB combination therapy for MDS patients.

It is common practice to detect cytogenetic changes in patients during the diagnosis of hematological malignancies to stratify risk and predict clinical outcomes, but due to the heterogeneity and complexity of the diseases, individuals differ in responses to chemotherapy, duration of remission, and recurrence although they have the same cytogenetic changes [52]. In recent years, genetic aberrations have significant practical value, and some have been used in National Comprehensive Cancer Network (NCCN) guidelines [53, 54]. Although the standard classification method based on IPSS-R includes chromosomal abnormalities, it lacks precise gene expression changes and mutations and cannot further individualize risk stratification for MDS patients. Thus, in this study, we constructed a new risk stratification consisting of *PDCD1*, *TIGIT*, *CD47*, *KIR3DL2* and the risk stratification based on the IPSS-R, which may provide more precise prognosis predictions for MDS. However, the small sample size of this study may have statistical bias, which may account for the lack of significant correlation between standard risk stratification based on IPSS-R and prognosis of patients in JUN-SMU dataset (Figure 5(e)). Moreover, there were a large number of low-risk patients in the GSE114922 dataset, while the proportion of high-risk patients in the JUN-SMU dataset was relatively high. Due to clinical sample limitations, we could not collect enough low-risk patients with sufficient follow-up time; thus, we will need to expand the sample size and include more samples from low-risk patients to verify our results in the future.

In conclusion, we demonstrated that high expression of *PDCD1*, *TIGIT*, *CD47*, and *KIR3DL2*, which are related to NK cells, is associated with poor OS for MDS patients, and co-expression of *PDCD1* and *TIGIT* might be the best OS predictor for MDS. Moreover, a new risk stratification paradigm consisting of *PDCD1*, *TIGIT*, *CD47*, and *KIR3DL2* expression and the standard risk stratification based on IPSS-R could provide more precise prognosis predictions for MDS. These findings might provide novel insight into prognosis stratification and designing combined targeted therapy for MDS.

## Figures and Tables

**Table 1:** Univariate and multivariate Cox regression analysis in MDS patients.

Variables	GSE114922 dataset				JNU-SMU dataset			
	Univariate Cox		Multivariate Cox		Univariate Cox		Multivariate Cox	
	HR (95% CI)	*P* value	HR (95% CI)	*P* value	HR (95% CI)	*P* value	HR (95% CI)	*P* value
*Gender*								
Female	Reference		Reference		Reference		Reference	
Male	2.88 (1.11, 7.48)	0.030	2.59 (0.95, 7.05)	0.063	3.39 (1.10, 10.48)	0.034	10.10 (2.08, 49.08)	0.004

*Age*								
≤60	Reference		Reference		Reference		Reference	
>60	1.33 (0.44, 4.00)	0.611	1.60 (0.48, 5.34)	0.45	0.99 (0.40, 2.47)	0.990	0.97 (0.34, 2.73)	0.951

*Risk stratification*								
Low risk	Reference		Reference		Reference		Reference	
Intermediate risk	3.60 (1.30, 9.98)	0.014	0.77 (0.21, 2.87)	0.694	2.78 (0.25, 31.30)	0.404	4.93 (0.27, 90.73)	0.283
High risk	4.57 (1.50, 14.01)	0.008	2.11 (0.56, 8.01)	0.272	2.94 (0.39, 22.24)	0.296	4.40 (0.35, 55.51)	0.252

*Risk stratification (estimated by total points)*								
Low risk	Reference		Reference		Reference		Reference	
Intermediate risk	3.90 (1.23, 12.35)	0.021	5.75 (1.28, 25.84)	0.023	1.44 (0.33, 6.39)	0.630	1.32 (0.20, 8.60)	0.770
High risk	12.47 (3.93, 35.56)	<0.001	10.83 (2.87, 40.93)	<0.001	5.01 (1.60, 15.68)	0.006	20.21 (3.90, >100)	<0.001

## Data Availability

The raw data used and/or analyzed during the current study may be available from the corresponding authors upon reasonable request.

## Supplementary Materials

Supplementary Materials Figure S1: overall survival (OS) analysis of CTLA4 and KLRC1 in MDS patients in the GSE114922 dataset. (A) The optimal cutoff value (upper) and Kaplan–Meier curve (bottom) of CTLA4. (B) The optimal cutoff value (upper) and OS analysis (bottom) of KLRC1. Figure S2: the optimal cutoff values of PDCD1, TIGIT, CD47, and KIR3DL2 in the GSE114922 (A–D) and the JNU-SMU (E–H) datasets. Figure S3: co-expression patterns of PDCD1, TIGIT, CD47, and KIR3DL2 were related to poor OS in MDS patients in the GSE114922 dataset. Figure S4: the optimal cutoff value of the total points in the monogram model was provided by X-tile software (version 3.6.1). Table S1: clinical information of the MDS patients. Table S2: primers for qRT-PCR. Table S3: points and OS rates in the nomogram model.
