# Supplementary material for: High Co-Expression of PDCD1/TIGIT/CD47/KIR3DL2 in Bone Marrow Is Associated with Poor Prognosis for Patients with Myelodysplastic Syndrome
Source: J Oncol. 2023 Feb 8;2023:1972127. doi: 10.1155/2023/1972127 (PMC9931467; doi:10.1155/2023/1972127)
Supplement: Supplementary Materials — Figure S1: overall survival (OS) analysis of CTLA4 and KLRC1 in MDS patients in the GSE114922 dataset. (A) The optimal cutoff value (upper) and Kaplan–Meier curve (bottom) of CTLA4. (B) The optimal cutoff value (upper) and OS analysis (bottom) of KLRC1. Figure S2: the optimal cutoff values of PDCD1, TIGIT, CD47, and KIR3DL2 in the GSE114922 (A–D) and the JNU-SMU (E–H) datasets. Figure S3: co-expression patterns of PDCD1, TIGIT, CD47, and KIR3DL2 were related to poor OS in MDS patients in the GSE114922 dataset. Figure S4: the optimal cutoff value of the total points in the monogram model was provided by X-tile software (version 3.6.1). Table S1: clinical information of the MDS patients. Table S2: primers for qRT-PCR. Table S3: points and OS rates in the nomogram model. [file 1972127.f1.docx]

**Supplementary Information**


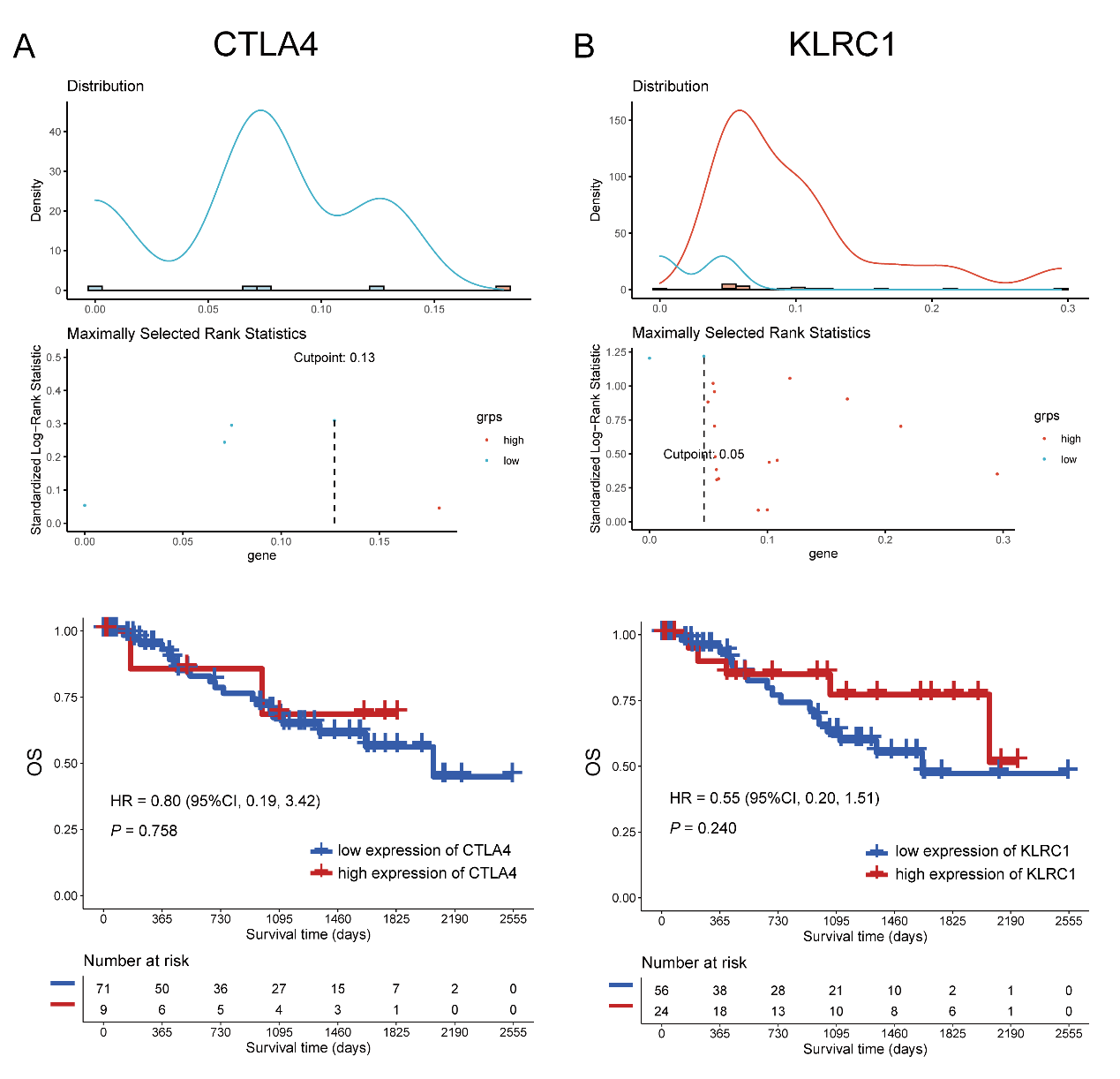


**Figure S1**. Overall survival (OS) analysis of *CTLA4* and *KLRC1* in MDS patients in the GSE114922 dataset. (A) The optimal cutoff value (upper) and Kaplan-Meier curve (bottom) of *CTLA4*. (B) The optimal cutoff value (upper) and OS analysis (bottom) of *KLRC1*.


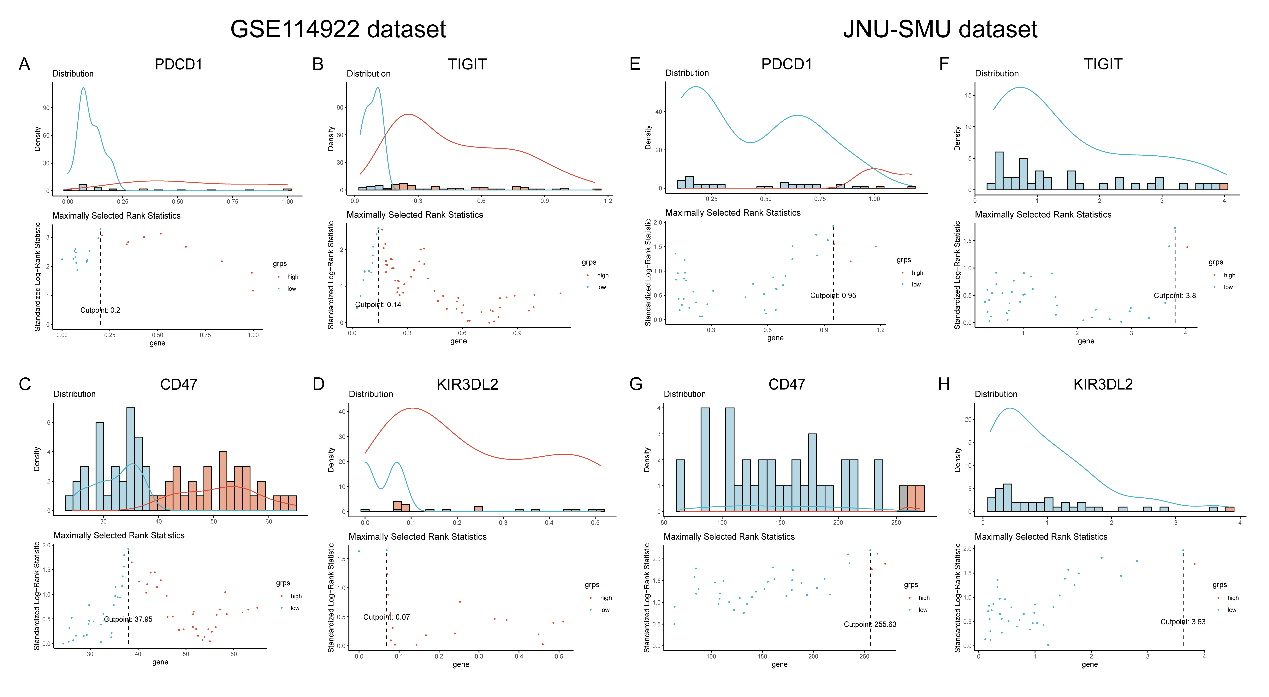


**Figure S2**. The optimal cutoff values of *PDCD1*, *TIGIT*, *CD47*, and *KIR3DL2* in the GSE114922 (A-D) and the JNU-SMU (E-H) datasets.


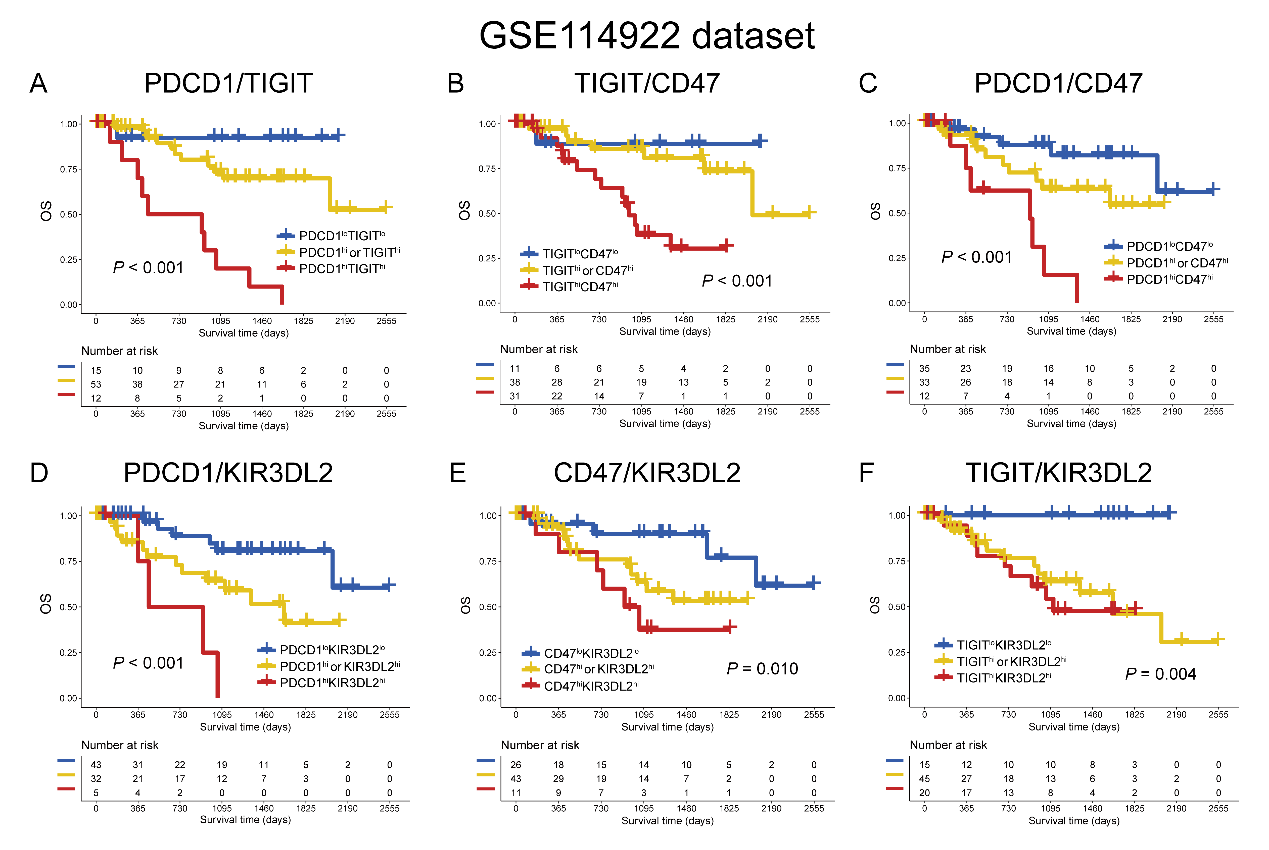


**Figure S3**. Co-expression patterns of *PDCD1*, *TIGIT*, *CD47*, and *KIR3DL2* were related to poor OS in MDS patients in the GSE114922 dataset.


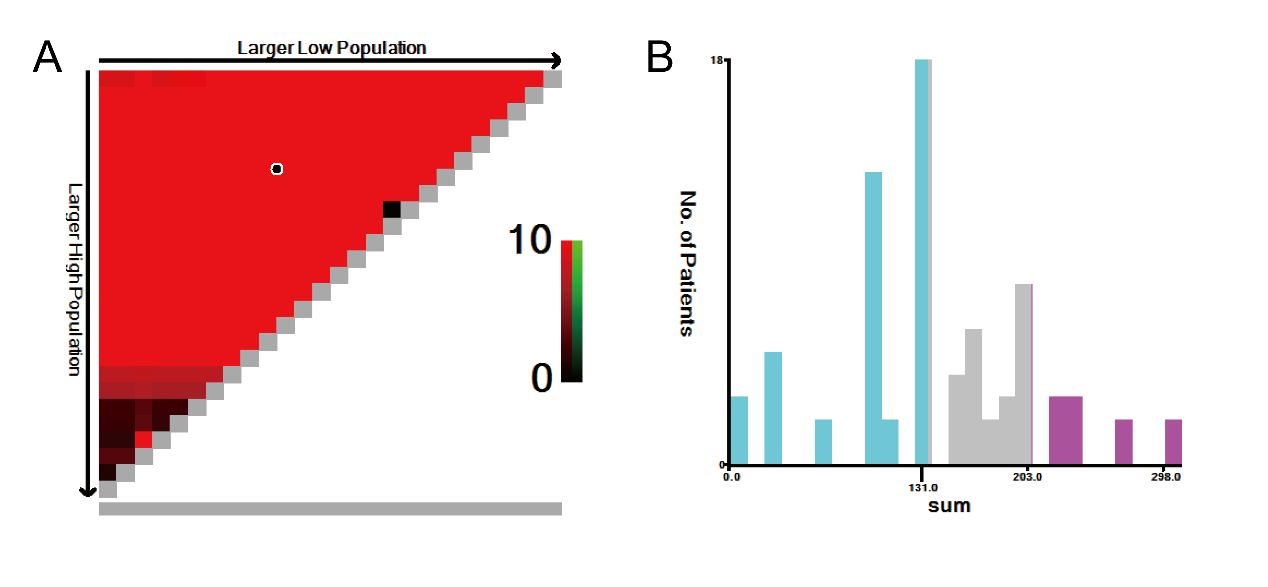


**Figure S4**. The optimal cutoff value of the total points in the monogram model was provided by X-tile software (version 3.6.1).

| **Variables** | **GSE114922** | **JNU-SMU** |
| --- | --- | --- |
| Number | 80 | 46 |
| Sample source | BM | BM |
| Cancer type, n (%) |  |  |
| MDS | 80 (100) | 41 (89.13) |
| sAML | 0 (0) | 5 (10.87) |
| Gender, n (%) |  |  |
| Female | 33 (41.25) | 16 (34.78) |
| Male | 47 (58.75) | 30 (65.22) |
| Age, years, median (range) | 66 (26, 87) | 54 (24, 82) |
| Risk stratification by IPSS-R, n (%) |  |  |
| Low/very low | 38 (47.50) | 6 (13.04) |
| Intermediate | 19 (23.75) | 5 (10.87) |
| High/very high | 19 (23.75) | 32 (69.57) |
| Unknown | 4 (5) | 3 (6.52) |
| OS, days, median (range) | 838.5 (0, 2549) | 393 (27, 1418) |
| Status (alive / death), n | 58 / 22 | 24 / 22 |

**Table S1.** Clinical information of the MDS patients.

MDS, myelodysplastic syndrome; IPSS-R, revised international prognostic scoring system; OS, overall survival; sAML, secondary acute myeloid leukemia

| **Targets** | **Sequence 5’-3’** |
| --- | --- |
| β-actin-F | TTGTTACAGGAAGTCCCTTGCC |
| β-actin-R | ATGCTATCACCTCCCCTGTGTG |
| PDCD1-F | CCAGGATGGTTCTTAGACTCCC |
| PDCD1-R | TTTAGCACGAAGCTCTCCGAT |
| TIGIT-F | CCAGATTCCATTGCTTGGAG |
| TIGIT-R | ATTCCTCCTGTCCAGCTGA |
| CD47-F | AGAAGGTGAAACGATCATCGAGC |
| CD47-R | CTCATCCATACCACCGGATCT |
| KIR3DL2-F | CAACTTCTCCATCGGTCCCTTGATG |
| KIR3DL2-R | GTTTGACCACACGCAGGGCAG |

**Table S2.** Primes for qRT-PCR.

|  | **Points** | **Survival time** | **OS rate** | **Total points** |
| --- | --- | --- | --- | --- |
| *CD47* | | 1 year | 0.95 | 171 |
| low | 0 |  | 0.90 | 220 |
| high | 31 |  | 0.85 | 233 |
| *KIR3DL2* | |  | 0.80 | 250 |
| low | 0 |  | 0.70 | 276 |
| high | 28 |  | 0.60 | 295 |
| *PDCD1* | | 2 year | 0.95 | 97 |
| low | 0 |  | 0.90 | 136 |
| high | 64 |  | 0.85 | 159 |
| *TIGIT* | |  | 0.80 | 177 |
| low | 0 |  | 0.70 | 202 |
| high | 100 |  | 0.60 | 222 |
| Risk stratification | |  | 0.50 | 238 |
| low | 0 |  | 0.40 | 253 |
| intermediate | 35 |  | 0.30 | 268 |
| high | 75 |  | 0.20 | 284 |
|  |  |  | 0.10 | 303 |
|  |  | 3 year | 0.95 | 52 |
|  |  |  | 0.90 | 91 |
|  |  |  | 0.85 | 115 |
|  |  |  | 0.80 | 132 |
|  |  |  | 0.70 | 157 |
|  |  |  | 0.60 | 177 |
|  |  |  | 0.50 | 193 |
|  |  |  | 0.40 | 209 |
|  |  |  | 0.30 | 223 |
|  |  |  | 0.20 | 239 |
|  |  |  | 0.10 | 259 |
|  |  |  | 0.05 | 273 |

**Table S3.** Points and OS rates in nomogram model.
